# A systematic review comparing surveillance recommendations for the detection of recurrence following surgery across 16 common cancer types

**DOI:** 10.1136/bmjonc-2024-000627

**Published:** 2025-03-07

**Authors:** Hannah Harrison, Bhumi K Shah, Faris Khan, Carley Batley, Chiara Re, Sabrina H Rossi, Georgia Stimpson, Eamonn Gilmore, Eleanor White, Sofia Kler-Sangha, Aufia Espressivo, Z Sienna Pan, Tanzil Rujeedawa, Benjamin W Lamb, Laura Succony, Shi Lam, Bincy M Zacharia, Rebecca Lucey, Alexander J P Fulton, Dimana Kaludova, Anita Balakrishnan, Juliet A Usher-Smith, Grant D Stewart

**Affiliations:** 1 Department of Public Health and Primary Care, University of Cambridge, Cambridge, UK; 2 School of Clinical Medicine, University of Cambridge, Cambridge, UK; 3 Department of Oncology, University of Cambridge, Cambridge, UK; 4 Department of Surgery, University of Cambridge, Cambridge, UK; 5 Unit of Urology, IRCCS San Raffaele Scientific Institute, Universita Vita Salute San Raffaele, Milano, Italy; 6 Independent Researcher; 7 Department of Urology, Barts Health NHS Trust, London, UK; 8 Barts Cancer Institute, Queen Mary University of London, London, UK; 9 Department of Urology, University College London Hospitals NHS Foundation Trust, London, UK; 10 Department of Thoracic Oncology, Royal Papworth Hospital NHS Foundation Trust, Cambridge, UK; 11 Hepato-Pancreato-Bilary Surgery, Cambridge University Hospitals NHS Foundation Trust, Cambridge, UK; 12 Precision Breast Cancer Institute, Department of Oncology, University of Cambridge, Cambridge, UK; 13 Department of Oncology, Cambridge University Hospitals NHS Foundation Trust, Cambridge, UK; 14 Cancer Research UK Cambridge Centre, University of Cambridge, Cambridge, UK; 15 Department of Surgery, Cambridge University Hospitals NHS Foundation Trust, Cambridge, UK

**Keywords:** Medical oncology, Health economics

## Abstract

**Objectives:**

Identify and compare guidelines making recommendations for surveillance to detect recurrence in 16 common solid cancers after initial curative treatment in asymptomatic patients.

**Methods and analysis:**

We conducted a systematic review, combining search results from two electronic databases, one guideline organisation website (NICE), expert consultation and manual searching. Screening and data extraction were carried out by multiple reviewers. We collected data from each guideline on recommendations for surveillance and the use of risk stratification. Findings were compared between cancer types and regions. Text mining was used to extract statements on the evidence for surveillance. A protocol was published on PROSPERO in 2021 (CRD42021289625).

**Results:**

We identified 123 guidelines across 16 cancer types. Almost all guidelines (n*=*115, 93.5%) recommend routine surveillance for recurrent disease in asymptomatic patients after initial treatment. Around half (n=59, 51.3%) recommend indefinite or lifelong surveillance. The most common modality of surveillance was cross-sectional imaging. Risk stratification of frequency, length and mode of surveillance was widespread, with most guidelines (n*=*92, 74.8%) recommending that surveillance be adapted based on patient risk. More than a third (n*=*50, 39.0%) gave incomplete or vague recommendations. For 14 cancers, we found statements indicating there is no evidence that surveillance improves survival.

**Conclusion:**

Although specific details of follow-up schedules vary, common challenges were identified across cancer types. These include heterogenous recommendations, vague or non-specific guidance and a lack of cited evidence supporting the use of surveillance to improve outcomes. Evidence generation in this area is challenging; however, increased availability to linked health records may provide a way forward.

**PROSPERO registration number:**

CRD42021289625.

**WHAT IS ALREADY KNOWN ON THIS TOPIC**

- Surveillance of asymptomatic patients for recurrence is common after curative surgery for cancer.
- The goal of surveillance is to improve early detection rates of recurrent disease and hence improve survival.
- For many cancers, there are multiple guidelines which make recommendations about follow-up care in cancer patients.

**WHAT THIS STUDY ADDS**

- Almost all guidelines for the 16 included solid cancers recommend surveillance after curative surgery, in many cases, this has an indefinite or lifelong duration.
- Recommendations are often vague or non-specific, which may lead to variation in the care delivered to patients.
- There is very little evidence presented in the guidelines that current recommendations for surveillance improve patient outcomes.

**HOW THIS STUDY MIGHT AFFECT RESEARCH, PRACTICE OR POLICY**

- We encourage guideline writers to consider the findings of this review when updating surveillance for recurrence recommendations, by providing clear and specific guidance that will limit variation in care and by acknowledging the lack of evidence in this area.
- We call for increased resources for research into surveillance for cancer recurrence and propose a framework (including observational data analysis, health economics and pragmatic trial designs) for a feasible approach.

## Introduction

 Large numbers of people are diagnosed with cancer every year (18 million in 2020), and incidence is expected to continue rising in the coming decades (28 million in 2024).^1^ In recent years, improved treatments have resulted in improved survival outcomes; in England, improvements to treatment have contributed to the 7.8% increase in 5-year survival rates between 2005 and 2016.^2^ Combined with improvements in the early detection of cancer, these trends result in increasing numbers of people living with and beyond a cancer diagnosis; it is estimated that by 2030, this will include four million people in the UK.^3^

Surgery is the first line of treatment for most solid non-metastatic cancers (either alone or in combination with adjuvant therapies). In England, 67% of treated cancers are managed surgically.^4^ After initial treatment, patients transition into follow-up care, which may include surveillance to detect recurrent cancer before the emergence of symptoms or further spread of disease. Intensive surveillance, for example, regular CT scans, is resource intensive for healthcare systems and places a high burden on patients. In a recent analysis of kidney cancer surveillance, it was estimated that 542 CT scans were required to detect one curable recurrence.^5^ Transition into follow-up care is often challenging for patients^6^,^8^; while surveillance can provide reassurance,^9^ the appointments, and waiting for the results, can also be a source of anxiety.^10 11^ A recent commentary on surveillance imaging highlighted the lack of resources available for research into the impact of surveillance, resulting in limited evidence of its efficacy.^12^

In this systematic review, we identified guidelines which include recommendations for surveillance to detect recurrence in asymptomatic individuals following surgical cancer treatment with curative intent. We included all 16 solid cancers in the list of 20 most common incident cancers in the UK^13^: breast, prostate, lung, bowel (colorectal), melanoma, kidney, head and neck, brain and central nervous system (brain hereafter), pancreas, bladder, uterus (endometrial), oesophagus, ovary, stomach (gastric), liver and thyroid. We compare identified guidelines to assess heterogeneity within and between cancer types, looking in detail at the types of surveillance recommended, the length of follow-up and the use of risk stratification. We also examine the evidence presented by the guidelines that surveillance improves outcomes for patients.

## Methods

We performed a systematic review following an a priori established study protocol (PROSPERO 2021 CRD42021289625).

Guidelines for inclusion were identified through a three-stage process. In the first stage, an electronic literature search of Medline, EMBASE and the National Institute for Health and Care Excellence (NICE) website search engine was performed (November 2021). Note that the NICE search engine captures publications from a range of guideline bodies (including but not limited to NICE guidelines). We included literature published in 2010–2021, using a combination of subject headings incorporating ‘cancer/neoplasm’ and ‘follow-up/surveillance’, limited to publications classified as guidelines (online supplemental appendix 1A).

We included guidelines that fulfilled the following criteria:

- Describe recommendations, strategies or information which can assist clinicians and patients to make decisions about routine surveillance (type, frequency, length) in asymptomatic adult patients after treatment with surgery with curative intent for one of the 16 included solid cancers.
- Produced by medical specialty associations, including relevant professional societies, public or private organisations, government agencies or healthcare providers at the state, national or international level.
- Freely available in print or electronic format in English.

Guidelines developed for specific populations (including groups with rare genetic conditions), less common cancer subtypes (<10% of incident cases of one of the listed solid cancers) or metastatic disease only were excluded. We only included the most recent guideline identified (for each cancer type) from each medical specialty association.

Two reviewers independently screened each publication identified in the searches by title and abstract to exclude clearly irrelevant results (BKS, FK, HH). If a definite decision to exclude could not be made, two reviewers independently screened the full text (BKS, FK, HH). Disagreements were resolved by discussion with the third reviewer.

In the second stage, clinical subject experts were consulted and asked to identify additional guidelines currently in use within their specialty. We consulted one UK-based clinical expert (identified through clinical networks) for each cancer type and sent them each a questionnaire with information about the guidelines identified in the first stage relating to their specialty. Additionally, manual checking of well-known guideline organisations (including the National Comprehensive Cancer Network (NCCN), European Society for Medical Oncology (ESMO) and American Society of Clinical Oncology (ASCO)) was carried out. All guidelines identified in the second stage were screened independently by two reviewers (HH/BKS).

In the third stage, to ensure the final set of results provided an up-to-date overview of the available documents, checks for updates to each guideline were carried out in April 2023 by one reviewer (HH).

A standardised data extraction form was developed by the study team and was piloted for the identified guidelines for one cancer type (kidney). For each included guideline, one reviewer extracted the data into the form, and a second reviewer checked the results (HH/BKS/FK/SHR/CR/LS/SL/BMZ/BWL/RL/AJPF/DK/EW/ZSP/EG/SKS/AE/TR/AB). Information about the population covered by the guideline, details of recommendations for routine surveillance in asymptomatic patients following curative treatment (including modality and length) and the use of risk-stratified surveillance were recorded. During data extraction, reviewers also identified any recommendations for surveillance that were vague, incomplete or non-specific. A narrative synthesis approach was used to identify similarities and differences between cancer types and regions.

To identify statements in the guidelines describing evidence that surveillance affects clinical outcomes, a text mining approach (online supplemental appendix 1B) was used to identify sentences in the guideline documents containing words relating to evidence (including ‘evidence’ and ‘data’) alongside words relating to follow-up or relevant outcomes (including ‘surveillance’, ‘follow-up’, ‘outcome’, ‘survival’ and ‘mortality’). The validity of this approach was tested by checking that statements on this topic identified by reviewers during data extraction were included in the results. The resulting sets of sentences were then manually screened.

Patients and members of the public have not been involved in this research study.

## Results

**Figure 1:**
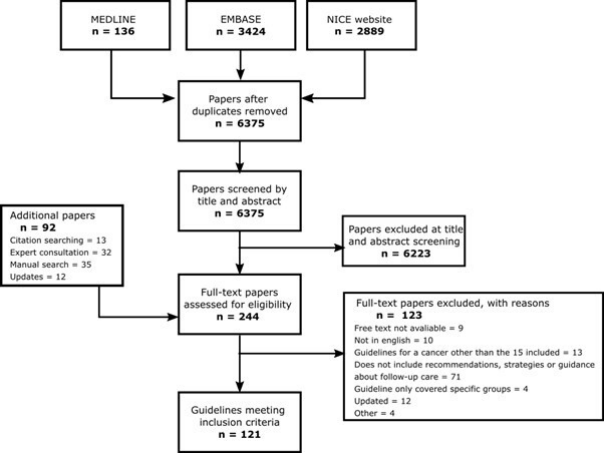
PRISMA flow diagram. PRISMA, Preferred Reporting Items for Systematic Reviews and Meta-Analyses.

After the removal of duplicates, the search of electronic databases and the NICE website identified 6385 publications, 6223 of which were excluded through title and abstract screening (n*=*162). A further 80 publications were identified through citation searching (n*=*13), consultation with experts (n*=*32) and manual checking of guideline organisations (n*=*35). These, 242 publications in total, were screened by full text, of which 121 were found to meet the review criteria. Five guidelines were replaced by an update from the same guideline body in April 2023. Two guidelines cover two cancer types (gastric and oesophageal), so we subsequently refer to 123 included guidelines. A PRISMA flow chart (figure 1), a list of included studies (online supplemental table S1a) and a list of studies excluded in full-text screening (online supplemental table S1b) are provided.

At least three guidelines were identified for each of the included cancer types (table 1, onlinesupplemental tables S2  S3a). The largest number of guidelines were identified for colorectal cancer (n*=*18, 14.6%) and the fewest for brain cancer (n*=*3, 2.4%). The identified guidelines were produced by 51 medical specialty organisations: well-known cancer guideline organisations (including NCCN (n*=*16, 13.0%), ESMO (n*=*16, 13.0%) and ASCO (n*=*6, 4.9%)); specialty organisations for specific cancer types (including the European Association of Urology (EAU) (n*=*3, 2.4%) and American Thyroid Association (ATA) (n*=*1, 0.81%)); and from national organisations providing guidance for healthcare systems (including NICE (n*=*12, 9.8%) and Cancer Council Australia (CCA) (n*=*3, 2.4%)). The guidelines covered a variety of geographic regions (online supplemental table S3b); however, the vast majority gave recommendations for populations in Europe (n*=*59, 48.0%), including 20 (16.3%) from the UK, and North America (n*=*46, 37.4%), of which the majority (n*=*41, 33.3%) were from the USA. We identified a small number of guidelines developed for populations in Asia (n*=*7, 5.7%), Oceania (n*=*5, 4.1%), South America (n*=*4, 3.3%) and the Middle East (n*=*1, 0.8%). Two guidelines were identified that did not relate to a specific region. No guidelines for countries or regions in Africa were identified.

**Table 1:** Summary of surveillance recommendations by cancer type

Cancer type	Number	Is surveillance to detect recurrence recommended?					Who is recommended for cross-sectional imaging as part of routine surveillance?				Fixed end point for surveillance
		Any (%)	Imaging (%)		Biomarkers (%)	Clinical appointment (%)					
			Cross-sectional	Localised			Everyone (%)	Some individuals (%)	Nobody (%)	Unclear (%)	
Overall	123	115 (93.5)	82 (66.7)	74 (60.2)	55 (44.7)	71 (57.7)	25 (20.3)	47 (38.2)	39 (31.7)	12 (9.8)	25 (20.3)
Bladder	10	10 (100)	10 (100)	10 (100)	9 (90.0)	2 (20.0)	1 (10.0)	9 (90.0)	–	–	1 (10.0)
Brain	3	3 (100)	3 (100)	–	–	2 (66.7)	3 (100)	–	–	–	–
Breast	9	9 (100)	2 (22.2)	9 (100)	–	7 (77.8)	–	2 (22.2)	7 (77.8)	–	4 (44.4)
Colorectal	18	18 (100)	16 (88.9)	16 (88.9)	13 (72.2)	11 (61.1)	7 (38.9)	7 (38.9)	2 (11.1)	2 (11.1)	4 (22.2)
Endometrial	4	4 (100)	2 (50.0)	–	–	4 (100)	–	2 (50.0)	2 (50.0)	–	3 (75.0)
Gastric	7	5 (71.4)	4 (57.1)	4 (57.1)	2 (28.6)	2 (28.6)	1 (14.3)	3 (42.9)	3 (42.9)	–	2 (28.6)
Head and neck	6	6 (100)	2 (33.3)	5 (83.3)	–	4 (66.7)	–	3 (50.0)	3 (50)	–	–
Kidney	7	7 (100)	7 (100)	5 (71.4)	4 (57.1)	3 (42.9)	7 (100)	–	–	–	1 (14.3)
Liver	5	5 (100)	4 (80.0)	–	3 (60.0)	1 (20.0)	4 (80.0)	–	–	1 (20.0)	1 (20.0)
Lung	6	6 (100)	5 (83.3)	–	–	5 (83.3)	–	–	1 (16.7)	5 (83.3)	–
Melanoma	15	15 (100)	14 (93.3)	12 (80.0)	6 (40)	14 (93.3)	–	13 (86.7)	1 (6.7)	1 (6.7)	6 (40.0)
Oesophageal	7	5 (71.4)	4 (57.1)	4 (57.1)	–	3 (42.9)	1 (14.3)	3 (42.9)	3 (42.9)	–	2 (28.6)
Ovarian	7	5 (71.4)	1 (14.3)	2 (28.6)	2 (28.6)	5 (71.4)	–	–	6 (85.7)	1 (14.3)	1 (14.3)
Pancreas	4	2 (50.0)	2 (50.0)	–	2 (50.0)	2 (50.0)	1 (25.0)	–	2 (50.0)	1 (25.0)	–
Prostate	8	8 (100)	–	–	8 (100)	3 (37.5)	–	–	8 (100)	–	–
Thyroid	7	7 (100)	6 (85.7)	7 (100)	6 (85.7)	3 (42.9)	–	5 (71.4)	1 (14.3)	1 (14.3)	–

### Recommendation for surveillance

Of the guidelines assessed, almost all (n*=*115, 93.5%) recommend at least some form of routine surveillance for recurrent disease in asymptomatic patients after initial surgery (table 1). Eight guidelines (covering four cancer types) recommend against routine surveillance for asymptomatic patients:

- Two European guidelines for ovarian cancer pre-dating the widespread use of the biomarker cancer antigen 125 (CA125).
- The two UK-based guidelines for oesophageal and gastric cancer.
- The two European guidelines for pancreatic cancer.

### Duration of surveillance

**Figure 2:**
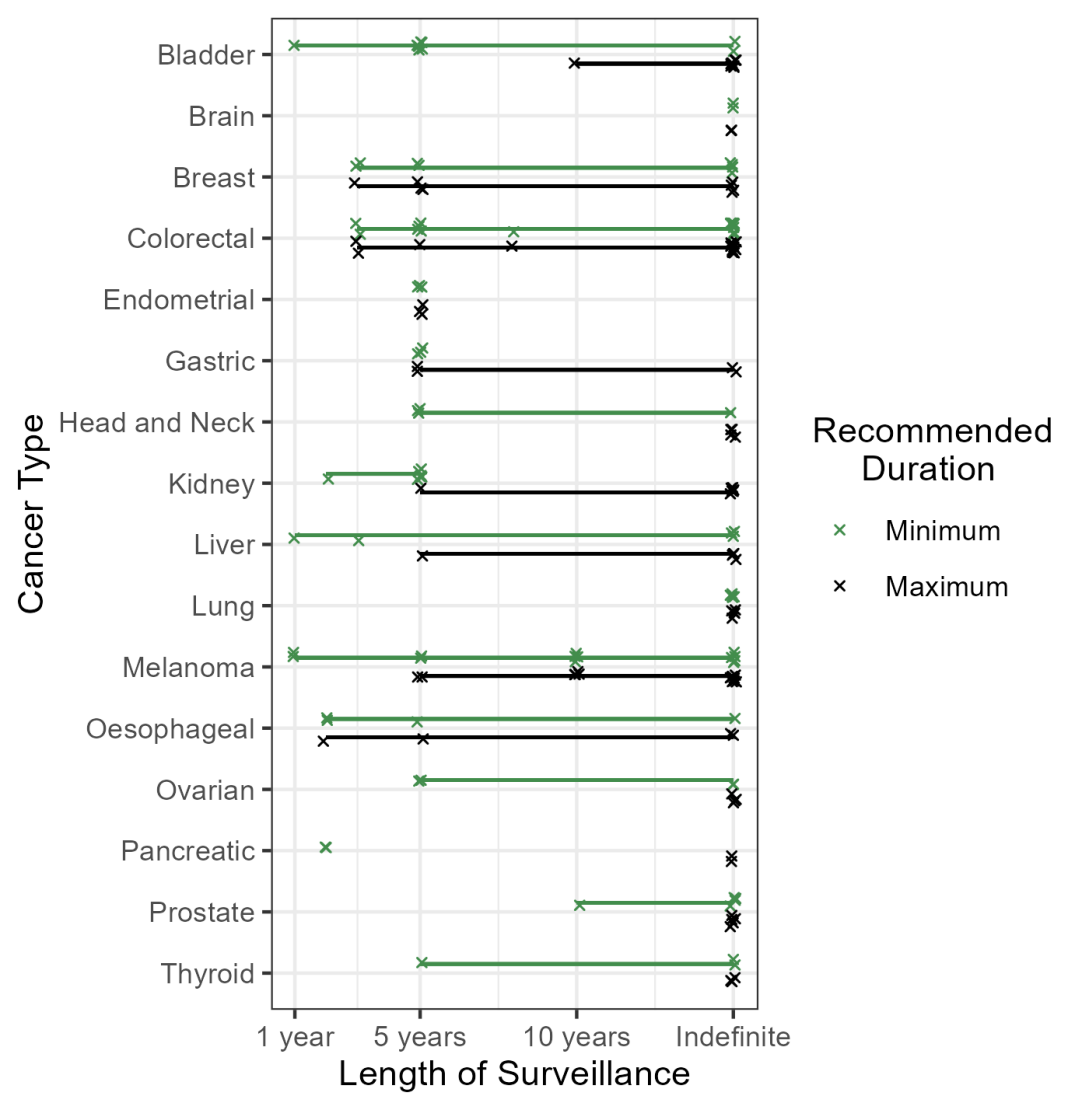
Recommendations for minimum and maximum durations of surveillance from all included guidelines by cancer type. Individual recommendations are shown with points, and lines give the range of (minimum and maximum) durations for each cancer.

Of the 115 guidelines which recommend some form of surveillance, most (n*=*81, 70.4%) do not recommend a fixed end point (figure 2, onlinesupplemental tables S2  S3a). Six explicitly recommend lifelong surveillance (including 3 of 15 melanoma guidelines) and 53 recommend that surveillance should be indefinite for all patients (including 5 of 6 lung cancer guidelines). There is no mention of the expected length of surveillance in 22 of the guidelines.

Of the remaining 34 guidelines, 25 recommend a fixed duration of surveillance for all patients. This includes three (75%) of the guidelines for endometrial cancer, four for breast cancer (44.4%) and six for melanoma (40%). Of these 25, most (n*=*19, 76.0%) recommend stopping surveillance for all patients by 5 years and the remainder recommend stopping by 10 years. No guidelines for brain, head and neck, lung, pancreas, prostate or thyroid cancers and only one guideline for bladder, kidney, liver and ovarian cancers give fixed durations for follow-up for all patients.

The nine remaining guidelines give a fixed duration for some patients and an indefinite (or lifelong) duration for others (using a risk-stratified approach). This includes seven guidelines for bladder cancer (70%), which advise adjusting the length of follow-up based on risk of recurrence. For example, the EAU guidelines recommend that those at lowest risk should stop follow-up after 5 years while those at highest risk should have lifelong follow-up.

### Modalities of surveillance

The most common surveillance modality is cross-sectional imaging—which includes in this analysis all non-localised imaging (table 1, online supplemental table S4a). This is recommended for at least some patients by 82 (66.7%) of the guidelines. There are three cancers (bladder, brain and kidney) for which all the identified guidelines recommend cross-sectional imaging as part of routine surveillance. There are seven further cancers in which most guidelines recommend this type of imaging for at least some asymptomatic patients during routine surveillance: melanoma (93.3%), colorectal (88.9%), thyroid (85.7%), lung (83.3%), liver (80%), oesophageal (57.1%) and gastric (57.1%). None of the prostate cancer and only one of the guidelines for ovarian cancer recommend the use of this type of imaging (instead relying mainly on prostate-specific antigen (PSA), or a combination of gynaecological exams and CA125 testing respectively).

There are differences in recommendations for the use of cross-sectional imaging for surveillance between different regions (onlinesupplemental tables S3bd). For colorectal, endometrial, gastric, head and neck, liver, lung, oesophageal, ovarian, pancreatic and thyroid cancers, the proportion of European guidelines recommending this type of imaging is lower than the proportion of North American guidelines making the same recommendation. For ovarian and pancreatic cancers, the only recommendations for cross-sectional imaging are made by guidelines from the USA (n*=*1, 14.3% and n*=*2, 50% respectively). For lung and thyroid cancers, guidelines from the UK are the only ones which do not make a recommendation for cross-sectional imaging. Overall, the proportion of the guidelines from North America recommending at least some cross-sectional imaging during follow-up is substantially higher (n*=*39, 86.7%) than European guidelines (n*=*35, 60.3%).

The most common modality of cross-sectional imaging is CT scans, recommended for use in surveillance in more than half of the identified guidelines (n*=*67, 54.5%) (online supplemental table S4a). Almost all guidelines for bladder (n*=*9, 90.0%), colorectal (n*=*15, 83.3%), kidney (n*=*7, 100%), liver (n*=*4, 80.0%) and lung (n*=*5, 83.3%) recommend using CT scans in surveillance for at least some patients; whereas, for prostate, breast and brain cancers, no guidelines recommend the use of CT scans (instead PSA testing, mammograms and MRI are recommended respectively). Five different cross-sectional imaging modalities are recommended across the 15 melanoma guidelines (CT scans (n*=*11), MRI (n*=*3), PET (n*=*4), multimodal (n*=*5) and X-ray (n*=*2)).

Localised imaging (or visualisation) techniques are also widely recommended for surveillance (n*=*74, 60.2%) (table 1, online supplemental table S4b). For breast, bladder and thyroid cancers, all identified guidelines recommend localised imaging for at least some of the individuals undergoing routine surveillance (mammography, cystoscopy and ultrasound respectively). In contrast, none of the guidelines for brain, endometrial, liver, lung, pancreatic or prostate cancer recommend imaging of this type. These cancers rely on cross-sectional imaging (brain, liver, lung and pancreatic), biomarkers (pancreatic and prostate), physical exams (endometrial, lung, pancreatic and prostate) or symptom monitoring (brain, lung and pancreatic).

10 cancer types have at least one guideline that recommends using a biomarker within routine surveillance (n*=*55, 44.7%) (table 1, online supplemental table S4c). Biomarkers are recommended in most guidelines for six cancer types: bladder (n*=*9, 90%), colorectal (n*=*13, 72.2%), kidney (n*=*4, 57.1%), liver (n*=*3, 60%), prostate (n*=*8, 100%) and thyroid (n*=*6, 85.7%). Carcinoembryonic antigen (CEA) is recommended for use in surveillance of recurrence by 13 (72.2%) colorectal cancer guidelines, all prostate cancer guidelines (n*=*8) recommend the use of PSA and nine guidelines (90%) for bladder cancer recommend the use of urine cytology. For ovarian cancer, two guidelines (28.6%) recommend the use of CA125 in surveillance for recurrence; however, two other guidelines explicitly recommend against its use in this context, citing lack of evidence.

Around 10% of guidelines (n*=*12, 9.8%) recommended a non-specific modality of surveillance. For example, recommendations that imaging should be part of routine surveillance without further details.

### Risk-stratification and surveillance

The use of risk stratification during follow-up after surgery for cancer is widely recommended, with 92 of the included guidelines (74.8%)—and at least one guideline for each of the 16 cancers—recommending that different groups of patients receive different routine surveillance (table 2, online supplemental table S3a). Notably, all guidelines for four cancer types (bladder, head and neck, kidney and thyroid) recommend the use of risk stratification.

**Table 2:** Summary of risk stratification recommendations by cancer type

Cancer type	Number	Is risk stratification recommended?						
		Any (%)	What is adapted?				How?	
			Eligibility (%)	Type (%)	Frequency (%)	Length (%)	Clinical risk factors (%)	Non-clinical risk factors (%)
Overall	123	92 (74.8)	6 (4.9)	64 (52)	62 (50.4)	32 (26)	83 (67.5)	42 (34.1)
Bladder	10	10 (100)	–	9 (90.0)	7 (70.0)	7 (70.0)	10 (100)	2 (20.0)
Brain	3	2 (66.7)	–	–	2 (66.7)	1 (33.3)	2 (66.7)	2 (66.7)
Breast	9	5 (55.6)	–	3 (33.3)	3 (33.3)	1 (11.1)	4 (44.4)	5 (55.6)
Colorectal	18	15 (83.3)	2 (11.1)	11 (61.1)	7 (38.9)	7 (38.9)	12 (66.7)	8 (44.4)
Endometrial	4	3 (75.0)	–	2 (50.0)	2 (50.0)	–	3 (75.0)	–
Gastric	7	5 (71.4)	1 (14.3)	3 (42.9)	3 (42.9)	2 (28.6)	5 (71.4)	3 (42.9)
Head and neck	6	6 (100)	–	3 (50.0)	1 (16.7)	1 (16.7)	4 (66.7)	4 (66.7)
Kidney	7	7 (100)	–	5 (71.4)	7 (100)	3 (42.9)	6 (85.7)	4 (57.1)
Liver	5	4 (80.0)	–	–	3 (60.0)	–	4 (80.0)	–
Lung	6	4 (66.7)	–	1 (16.7)	3 (50.0)	–	3 (50.0)	2 (33.3)
Melanoma	15	14 (93.3)	–	14 (93.3)	12 (80.0)	3 (20.0)	14 (93.3)	6 (40.0)
Oesophageal	7	4 (57.1)	1 (14.3)	4 (57.1)	3 (42.9)	3 (42.9)	4 (57.1)	1 (14.3)
Ovarian	7	2 (28.6)	–	1 (14.3)	2 (28.6)	1 (14.3)	2 (28.6)	–
Pancreas	4	1 (25.0)	–	1 (25.0)	1 (25.0)	–	1 (25.0)	1 (25.0)
Prostate	8	3 (37.5)	–	1 (12.5)	1 (12.5)	1 (12.5)	2 (25.0)	2 (25.0)
Thyroid	7	7 (100)	2 (28.6)	6 (85.7)	5 (71.4)	2 (28.6)	7 (100)	2 (28.6)

**Figure 3:**
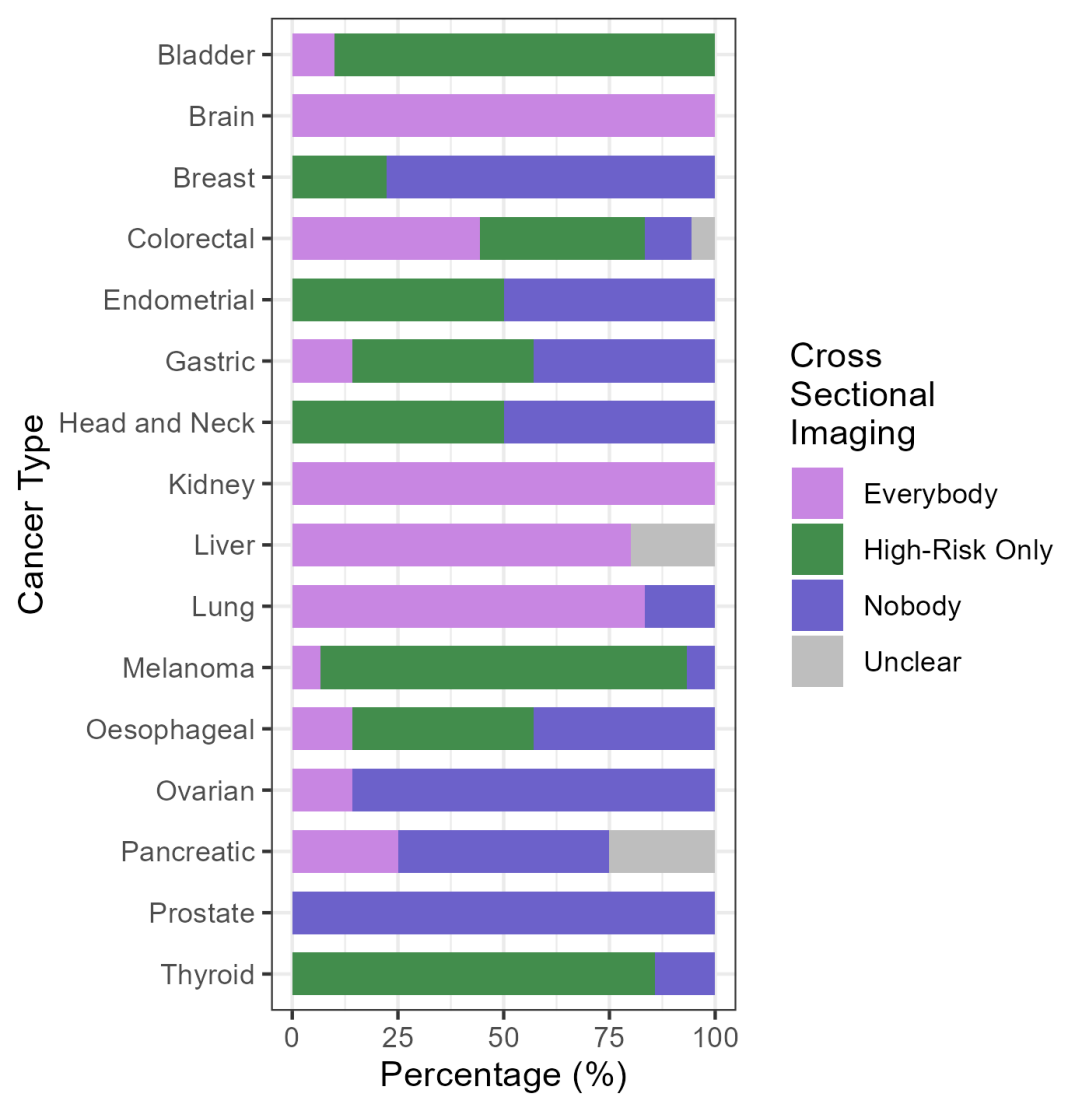
The proportion of guidelines recommending that everyone, only those at high risk of recurrence or nobody receives cross-sectional imaging as part of routine surveillance by cancer type. Unclear indicates that it was not possible to tell if cross-sectional imaging is recommended based on the information in the guideline (eg, if the modality of surveillance is not specified).

Many guidelines (n=63, 51.2%) recommend using stratification to determine the modality (or type) of surveillance. At least one guideline for 14 cancers (none for brain or liver cancer) stratifies in this way. Of the 82 guidelines that recommend the use of cross-sectional imaging, more than half (n=47, 57.3%) explicitly limit usage to higher-risk individuals (figure 3). This varies by cancer type: most of the guidelines for bladder (n*=*9, 90.0%), melanoma (n*=*13, 86.7%) and thyroid (n*=*6, 85.7%) recommend targeting the use of cross-sectional imaging, but two cancer types (brain and kidney) recommend some cross-sectional imaging for all patients.

At least one guideline for each cancer type recommends scheduling more frequent follow-up for patients at higher risk, including all seven kidney cancer guidelines and 12 (80.0%) of the melanoma guidelines. However, in 17 (out of 62), the details of the risk-adjusted surveillance schedules are incomplete. For example, three (out of seven) guidelines for gastric cancer recommend that higher-risk patients should have more frequent follow-up, but the recommended frequency is not stated.

Most of the guidelines recommending risk stratification (n*=*83, 90.2%), and at least one guideline for each cancer type, use clinical risk factors (ie, characteristics of the cancer being treated) to assess risk of recurrence and determine appropriate surveillance. Stage is the most widely mentioned clinical risk factor (n*=*63, 68.5%), used by guidelines for 14 cancer types, including almost all the guidelines for bladder (n*=*8, 80.0%), kidney (n*=*6, 85.7%) and thyroid cancers (n*=*6, 85.7%). Non-clinical risk factors, including demographic and lifestyle risk factors, are used in nearly half of the guidelines recommending risk stratification (n*=*42, 45.7%) and across 13 cancer types.

Nearly a third of guidelines (n*=*39, 31.7%) give an incomplete description of the recommended method of risk stratification—including at least one guideline for 11 cancer types. This is seen in seven melanoma guidelines (46.7%); clinicians are advised to classify patients as high-risk if they have ‘high risk pathologic features’ or ‘risk factors for melanoma development’.

### The evidence-basis for surveillance

Overall, 78 guidelines (63.4%) presented some evidence or assessed the evidence level for at least one aspect of their follow-up recommendation (onlinesupplemental tables S2  S3a); the remaining guidelines made recommendations based only on expert opinion (n*=*31, 25.2%) or did not state how the recommendations were reached (n=14, 11.4%).

Using a text mining approach, we identified 45 statements in guidelines for 14 cancer types which discuss the evidence that surveillance improves long-term outcomes for patients (online supplemental table S5). No statements of this type were identified in any of the guidelines for endometrial, liver or thyroid cancers.

We identified statements in two colorectal cancer guidelines asserting that intensive surveillance following surgery can improve overall survival (box 1). Conversely, we identified sentences in guidelines for two cancers (pancreatic, and head and neck) stating that there is no evidence for surveillance, in guidelines for three cancers (bladder, brain and pancreatic) that there is no benefit to surveillance and in guidelines for nine cancers (bladder, breast, colorectal, gastric, lung, melanoma, oesophagus, ovarian and prostate) that there is no evidence that surveillance improves outcomes (in most cases, survival is specified).

Box 1 **Statements on surveillance and survival (selected extracts)**

“…older meta-analyses have reported an improvement in overall survival in intensively surveilled populations.” (European Society of Gastrointestinal Endoscopy and European Society of Digestive Oncology, Colorectal Cancer Guideline, 2019)

“However, at this time, no data from prospective trials demonstrating the potential benefit of early detection of recurrent disease and its impact on OS are available.” (European Association of Urology, Bladder Cancer Guideline, 2021)

“There is currently no data demonstrating improvements in survival from routine surveillance.” (Cancer Care Ontario, Lung Cancer, 2014)

“Although there is no research showing that periodic imaging lengthens the OS, many countries’ guidelines recommended periodic imaging following curative resection of melanoma” (Japanese Dermatological Association, Melanoma Cancer Guideline, 2020)

Several guidelines discuss the link between surveillance, earlier diagnosis of recurrence and survival outcomes (box 2). Four guidelines for colorectal cancer state that surveillance improves early detection, while guidelines for melanoma and gastric cancers state that this improvement is expected. Guidelines for breast and lung cancer refer to evidence that early detection and early treatment of recurrence are associated with improved survival. However, guidelines for seven cancers (brain, colorectal, gastric, melanoma, oesophagus, ovarian and prostate) said that there is not any evidence that earlier detection of recurrence improves survival, and guidelines for three cancer types (kidney, ovarian and pancreatic) similarly claim that the earlier treatment of advanced disease does not improve survival (box 3). One guideline for ovarian cancer went further, citing evidence that treating recurrences early is associated with a decrease in quality of life.

Box 3 **Statements on early detection (or treatment) of recurrence and survival (selected extracts)**

“It is important to underscore the goal of surveillance in early-stage breast cancer, which is to detect early locoregional or contralateral recurrence, as early detection of breast cancer recurrence is correlated with improved survival.” (American College of Radiology, Breast Cancer Guideline, 2019)

“Nevertheless, there are no trials indicating that the earlier detection of recurrence and subsequent change in management improves outcomes.” (European Society for Medical Oncology, Prostate Cancer Guideline, 2020)

“The data suggest that treating recurrences early (based on detectable CA-125 levels in patients who are asymptomatic) is not associated with an increase in survival and is associated with a decrease in QOL” (National Comprehensive Cancer Network, Ovarian Cancer Guideline, 2023)

“…there is no evidence to show whether early detection leads to improved overall survival.” (National Institute for Health and Care Excellence, Gastric Cancer Guideline, 2018)

Box 2 **Statements on surveillance and early detection (selected extracts)**

“Intensive follow-up can detect recurrences earlier, thus surgical resection for curative intent is possible. However, this is not associated with improved survival.” (Cancer Council Australia, Colorectal Cancer Guideline, 2018)

“Although the hypothesis suggesting that regular monitoring reveals early detection of metastasis may be well founded, no randomised studies have demonstrated that early detection of metastases improves the overall survival.” (Swiss Guideline, Melanoma Guideline, 2016)

“A regular follow-up may allow investigation and treatment of symptoms, psychological support and early detection of recurrence, though there is no evidence that it improves survival outcomes” (European Society for Medical Oncology, Gastric Cancer Guideline, 2016)

Additionally, statements indicating a lack of evidence about the specific protocols or schedules for surveillance were identified in at least one guideline for 15 of the included cancer types (box 4, online supplemental table S5). No statements on this topic asserted that there was any evidence favouring one surveillance schedule over another.

Box 4 **Statements on evidence basis for specific surveillance protocols or schedules (selected extracts)**

“…there are no differences in overall survival with any of the different follow-up schemes… if the patient is guaranteed access to healthcare services in the presence of a symptom or sign of alarm.” (European Society for Medical Oncology, Breast Cancer Guideline, 2019)

“Local follow-up protocols are based more on historical practice than evidence and are often disease- rather than patient-centred.” (NICE, Head and Neck Cancer Guideline, 2016)

“However, the ideal monitoring intervals and methods require further research.” (Korean Liver Cancer Association–National Cancer Center Korea, Liver Cancer Guideline, 2019)

“There is no high-quality evidence to support one surveillance schedule for follow-up care of melanoma survivors, which results in great variability in guideline recommendations from different organizations.” (Cancer Care Ontario, Melanoma Cancer Guideline, 2015)

“Despite the fact that no randomised data exist to support any particular follow-up sequence or protocol, balancing patient needs, follow-up costs and burden is necessary…” (European Society for Medical Oncology, Bladder Cancer Guideline, 2019)

## Discussion

### Summary

In this multicancer review, we identified 123 guidelines for the surveillance of asymptomatic patients following curative surgery across 16 solid cancer types, finding that almost all guidelines recommend surveillance for recurrence in asymptomatic individuals (n*=*115, 93.5%). Where recommendations for surveillance were found, we noted substantial differences between guidelines both within and between cancer types, widespread inclusion of vague or non-specific recommendations and an almost complete absence of cited evidence that surveillance improves long-term patient cancer outcomes.

### Challenges identified

The heterogeneity observed between guidelines for the same cancer type was notable. Differences are observed in modality (five cross-sectional imaging modalities are recommended across the melanoma guidelines) and scheduling (the recommended length of surveillance for oesophageal cancer ranges from 2 years to indefinite) of surveillance, as well as in the use of risk stratification. There are notable exceptions to this such as: the use of PSA and cystoscopy is recommended in all the guidelines for prostate and bladder cancers respectively. Some of the heterogeneity can be explained by changing clinical practice over time (such as more widespread use of CA125 in recent ovarian cancer guidelines) or geographical variations in clinical practice. Across several cancer types, cross-sectional imaging is less widely recommended in Europe (particularly UK guidelines) compared with North America. However, there are also differences in recommendations between guidelines in the same region for the same cancer type; there are four guidelines from the USA for prostate cancer, two recommend more surveillance for those at higher risk of recurrence while the other two recommend the same surveillance for everyone. Further variation is seen in the assessments of the quality of the evidence, around a third (37%) of guidelines presented information about the level of evidence. Where the evidence level was given, we identified more than 20 different frameworks and widespread heterogeneity aspects of surveillance assessed (eg, some guidelines report on evidence that a certain imaging modality can identify recurrence while others discuss the evidence that a specific schedule can improve patient outcomes). Variation in the recommendations for follow-up care has been previously identified in reviews of guidelines conducted for prostate cancer^14^ and melanoma,^15^ in 2009 and 2015 respectively, corroborating our findings and suggesting this is an ongoing challenge.

We found several areas in which recommendations are frequently vague and non-specific. Many guidelines (n*=*53) recommend indefinite surveillance and others (n*=*22) do not mention the expected length of the recommended surveillance; decision-making about ending surveillance is left to individual clinicians. Patients not eligible for further treatment (for example, because they are too frail) are unlikely to see any benefit from continued surveillance for the early detection of recurrence. We also found that many guidelines recommending risk stratification did not clearly define the risk factors they suggest using and others gave only vague details of how surveillance schedules should differ between high and low-risk groups. This allows for variability in their interpretation by clinicians, likely to lead to variation in care. Given the limited evidence for the efficacy of any specific surveillance schedule, the impact of this on patient outcomes is not known. However, a standardised approach to surveillance is desirable, especially as variation in care has been identified as a key concern for patients receiving cancer follow-up care.^8 11 16^ Lack of clear definitions of risk factors was also seen in a previous review of follow-up guidelines for melanoma.^15^

Variation in the recommendations and a lack of specific details are likely connected to the limited evidence that surveillance improves patient outcomes, mentioned in guidelines for almost all the cancer types. The reported lack of evidence is particularly concerning given the widespread use of surveillance to detect recurrence. Many guidelines recommend lifelong or indefinite surveillance which presents a significant burden for both patients and healthcare systems. We highlight the frequent use of cross-sectional imaging, which typically involves exposure to radiation and uses valuable resources (equipment and trained healthcare professionals). The use of risk stratification, seen across all included cancer types, is a reasonable method to distribute resources among patients according to need, ideally improving rates of detection in high-risk patients and reducing burden for low-risk patients. However, we did not find any statements in guidelines discussing any evidence of the effect of a risk-stratified schedule on patient outcomes. Further, we only identified one guideline in which evidence of the potential harms of surveillance was mentioned (the NCCN guideline for ovarian cancer notes that surveillance is associated with decreased quality of life). Previous reviews of colorectal cancer^17^ and melanoma^15^ follow-up guidelines found a lack of evidence for the delivery of effective care and a need for research examining the benefits and costs for different follow-up strategies respectively. Patients have previously identified a lack of transparency^11^ and unmet information needs^7 9^ in follow-up care; we encourage clinicians to discuss the uncertainty around the benefits of many surveillance protocols with their patients to promote informed decision making.

### Strengths and weaknesses

This is the first systematic review of follow-up guidelines for multiple cancer types, providing a comprehensive overview of this area of cancer care with the use of a rigorous search strategy (combining electronic databases, grey literature, consultation with subject experts and manual searching) to ensure good coverage. This approach was required as, while publications from some guideline bodies were included in electronic databases (ESMO routinely publish guidelines in the well-indexed Annals of Oncology), others were only identifiable via their own websites (such as NCCN guidelines). The decision to limit this review to English language guidelines has led to an over-representation of guidelines from English-speaking countries (including the UK, USA and Canada) and from international guideline bodies which publish in English (such as ESMO). It is our view that this approach was sufficient to provide an overview of this topic—but results should be interpreted taking this into consideration. This body of literature is constantly evolving, and it is challenging to provide an up-to-date overview of such a large set of guidelines; we acknowledge that updates to a small number of guidelines may have been published since we updated our search in 2023. Any updates are unlikely to affect the finding of this review and the overarching challenges facing this field.

The use of a text mining approach to identify statements about evidence for surveillance in this review provided a pragmatic option for systematically identifying relevant sentences across many lengthy documents. While data extraction focused on subsections of documents discussing follow-up, this allowed for the inclusion of text from throughout the guidelines. However, relevant statements may have been missed due to the formatting of the guideline documents or if they used language not captured by the search terms.

Researchers interested in exploring this topic further—for example, conducting more in-depth reviews of individual cancer types, investigating in more detail the specific schedules of follow-up recommended or carrying out more detailed assessments of the evidence provided in the guidelines—may find the full list of included guidelines (including date, guideline body, URL) and the detailed extraction summary table (including details of the population, surveillance modalities and risk stratification methods) provided in the supplementary materials (onlinesupplemental tables S1a  S2) useful resources.

### Future research

We recommend that guideline bodies consider the findings of this review when updating their recommendations (with a focus on providing clear, specific advice that will help limit variation in care); we acknowledge that this is challenging while evidence in this area is lacking. Therefore, we take this opportunity to call for an increase in resources for research to address the lack of evidence that surveillance is beneficial to patients and a good use of healthcare resources. Although most included guidelines recommend lengthy surveillance, many guidelines acknowledge that there is little evidence that this improves long-term outcomes for patients. As recently highlighted, this is in stark contrast to the rigorous evidence required to offer drug-based interventions to patients.^12^ The long timeframes required and potential difficulties in patient recruitment make trials of alternative surveillance schedules prohibitively expensive and complex. Some studies, both observational^18^ and trial-based^19^ (for melanoma and colorectal cancer respectively), have had success in generating evidence around optimal surveillance schedules; an ongoing UK-based multicentre trial^20^ aims to produce evidence about the effect of follow-up on survival in patients after surgery for gastric cancer. However, it is difficult to design a trial when so many aspects of surveillance (modality, frequency, length, risk stratification method) are poorly understood. Ideally, studies would not only assess the ability of surveillance to improve early detection and verify that this is linked to improved survival outcomes but also consider questions about how best to implement risk stratification and the optimal surveillance modalities and schedules for patients at differing risk levels.

We encourage researchers to make use of existing data and to explore a range of methodologies to tackle the challenges and unknowns in this area. As population-scale health data, with many years of retrospective linkage, becomes more widely available,^21 22^ this may be a valuable resource for identifying patients who have received curative treatment for a primary cancer, tracking patient trajectory through follow-up and investigating the long-term association between surveillance and survival. Health economics approaches could be used to improve understanding of the costs of current surveillance to both patient and healthcare systems and probabilistic sensitivity analysis used to identify priority areas for research and data collection.^23^ Optimised surveillance strategies developed using observational data could then be used to inform the design of trials. Feasibility studies and pragmatic trials, designed to explore the acceptability and real-world clinical application of alternative surveillance strategies, may provide a suitable starting point, before progressing to more expensive randomised controlled trials. These should be designed to include patient-focused outcomes such as cancer anxiety, worry or fear, and quality of life, where current evidence is particularly poor.

## Supplementary material

10.1136/bmjonc-2024-000627 Supplementary file 1

10.1136/bmjonc-2024-000627 Supplementary file 2

10.1136/bmjonc-2024-000627 Supplementary file 3

10.1136/bmjonc-2024-000627 Supplementary file 4

10.1136/bmjonc-2024-000627 Supplementary file 5

10.1136/bmjonc-2024-000627 Supplementary file 6

10.1136/bmjonc-2024-000627 Supplementary file 7

10.1136/bmjonc-2024-000627 Supplementary file 8

10.1136/bmjonc-2024-000627 Supplementary file 9

10.1136/bmjonc-2024-000627 Supplementary file 10

10.1136/bmjonc-2024-000627 Supplementary file 11

10.1136/bmjonc-2024-000627 Supplementary file 12

## Data Availability

Data are available upon reasonable request.
