# Supplementary material for: A systematic review comparing surveillance recommendations for the detection of recurrence following surgery across 16 common cancer types
Source: BMJ Oncol. 2025 Mar 7;4(1):e000627. doi: 10.1136/bmjonc-2024-000627 (PMC12164354; doi:10.1136/bmjonc-2024-000627)
Supplement: Supplementary file 1 [file bmjonc-4-1-s001.docx]

**A: Search Strategy (Embase, Medline and NICE)**

**Embase** <1974 to 2021 November 04> - search run on 05/11/2021

1 exp solid malignant neoplasm/ 1791871

2 (cancer* or neoplasm* or carcinom* or tumo?r or melanom*).mp. [mp=title, abstract, heading word, drug trade name, original title, device manufacturer, drug manufacturer, device trade name, keyword heading word, floating subheading word, candidate term word] 5622856

3 exp cancer prognosis/ or exp monitoring/ or exp follow up/ or exp risk assessment/ or exp cancer risk/ 3424724

4 (follow?up or suv?llance or manag* or stratif* or prognos*).mp. [mp=title, abstract, heading word, drug trade name, original title, device manufacturer, drug manufacturer, device trade name, keyword heading word, floating subheading word, candidate term word] 4406867

5 1 or 2 5695146

6 3 or 4 6815683

7 5 and 6 1708321

8 exp practice guideline/ 618101

9 7 and 8 59488

10 exp clinical trial/ or cross-over studies/ or double-blind method/ or random allocation/ or randomized controlled trials as topic/ or single-blind method/ 1879447

11 epidemiology/ or exp case control study/ or cohort analysis/ or case study/ or longitudinal study/ or retrospective study/ or prospective study/ or observational study/ or correlation study/ or cross-sectional study/ 3250356

12 10 or 11 4763603

13 9 not 12 39133

14 guideline.ti,ab. 104343

15 13 and 14 3998

16 limit 15 to yr="2010 -Current" 3424

Ovid **MEDLINE**(R) and Epub Ahead of Print, In-Process, In-Data-Review & Other Non-Indexed Citations, Daily and Versions(R) <1946 to November 04, 2021> - search run on 05/11/2021

1 exp Neoplasms/ 3565965

2 (cancer* or neoplasm* or carcinom* or tumo?r or melanom*).mp. [mp=title, abstract, original title, name of substance word, subject heading word, floating sub-heading word, keyword heading word, organism supplementary concept word, protocol supplementary concept word, rare disease supplementary concept word, unique identifier, synonyms] 4278744

3 exp risk assessment/ or exp cancer risk/ or exp Prognosis/ 1975935

4 (follow?up or suv?llance or manag* or stratif* or prognos*).mp. [mp=title, abstract, original title, name of substance word, subject heading word, floating sub-heading word, keyword heading word, organism supplementary concept word, protocol supplementary concept word, rare disease supplementary concept word, unique identifier, synonyms] 2720291

5 1 or 2 4699608

6 3 or 4 3866412

7 5 and 6 1081557

8 limit 7 to guideline 922

9 limit 8 to yr="2010 -Current" 136

**NICE website search** (https://www.nice.org.uk/) carried out on 04/11/2021

“cancer” OR “carcinoma” OR “neoplasm” OR “tumour” OR “melanoma” OR “lymphoma” limited to results tagged as Guidance (2889 results).

**B: Text Mining Search terms**

*Search terms*

patt_1 <- "evidence*|proof*|prove*|data*"

patt_2 <- "surveillance|survival|recurr*|relapse|death|mortality|follow-up|interval*|sequence*|protocol*|scheme*|frequency|length|timing|schedule|outcome|monitoring"

patt_3 <- "diag*|adjuv*|chemo*|toxic*|preoper*|metast*|margin*|germline|somatic|tamoxifen|sorafenib|tissue|inhibitor|radio*|biops*|dos*|drain|bevacizumab|platinum*|implant*|fertil*|ontario"

*Filter*

(patt1 AND patt2) NOT patt 3
